# SPADE: Spatial transcriptomics and pathology alignment using a mixture of data experts for an expressive latent space

**DOI:** 10.1016/j.media.2026.104197

**Published:** 2026-07-09

**Authors:** Ekaterina Redekop, Mara Pleasure, Zichen Wang, Vedrana Ivezic, Kimberly E. Flores, Benjamin Emert, Anthony Sisk, William Speier, Corey W. Arnold

**Affiliations:** a Biomedical AI Research Lab, University of California, 924 Westwood Blvd, Los Angeles, 90024, CA, United States; b Department of Radiological Sciences, University of California, Los Angeles, CA, United States; c Department of Pathology, University of California, Los Angeles, CA, United States; d Department of Bioengineering, University of California, Los Angeles, CA, United States; e Medical Informatics Home Area, University of California, Los Angeles, CA, United States

**Keywords:** Digital pathology, Spatial transcriptomics, Mixture of data experts, Contrastive learning, Self supervision

## Abstract

The rapid growth of digital pathology and advances in self-supervised deep learning have enabled the development of foundational models for various pathology tasks across diverse diseases. While multimodal approaches integrating diverse data sources have emerged, a critical gap remains in the comprehensive integration of whole-slide images (WSIs) with spatial transcriptomics (ST), which is crucial for capturing critical molecular heterogeneity beyond standard hematoxylin & eosin (H&E) staining. We introduce SPADE, a foundation model that integrates histopathology with ST data to guide image representation learning within a unified framework, in effect creating an ST-informed latent space. SPADE leverages a mixture-of-data experts technique, where experts are created via two-stage imaging feature-space clustering using contrastive learning to learn representations of co-registered WSI patches and gene expression profiles. Pre-trained on the comprehensive HEST-1k dataset, SPADE is evaluated on 20 downstream tasks, demonstrating significantly superior few-shot performance compared to baseline models, highlighting the benefits of integrating morphological and molecular information into one latent space. Code and pretrained weights are available at https://github.com/uclabair/SPADE.

## 1. Introduction

High-resolution whole slide images (WSIs) have propelled the development of powerful deep learning foundation models in computational pathology, demonstrating robust performance across diverse tissue types and tasks (Wang et al., 2022; Li et al., 2021; Saednia et al., 2022; Howard et al., 2023). These models are typically trained using self-supervision, enabling learning from large unlabeled datasets and producing embeddings robust to institutional variations, including differences in staining procedures and other image-quality factors (Wang et al., 2024; Chen et al., 2024, 2022; Xu et al., 2024).

By visually capturing cellular arrangement, WSIs enable the study of spatial organization and disorganization of cells in tissues, characterizations that are especially relevant in cancer research (Moffitt et al., 2022; Moses and Pachter, 2022). In clinical settings, WSIs are commonly stained with hematoxylin & eosin (H&E), a two-color stain that highlights nuclei and cytoplasm but offers a limited view of molecular-level heterogeneity (Lee et al., 2023). As tumor tissues are known to exhibit high variability within and across patients, deciphering the heterogeneity at the molecular level is critical for improving deep learning applications that can more precisely inform diagnosis, treatment, and prognosis (Rao et al., 2021; Xun et al., 2023). While H&E provides crucial morphological insights, its inability to capture molecular heterogeneity limits its utility in fully characterizing tissue complexity.

Spatial transcriptomics addresses this gap by providing spatially resolved gene expression data, allowing for additional molecular context for a given tissue specimen. Although both ST and H&E data have independently proven useful in various applications, their combined potential for creating a more comprehensive representation learning framework remains unexplored. To this end, we introduce SPADE, a vision–ST foundation model that uses a mixture of experts, each trained via contrastive learning, to unify ST data and H&E images to produce slide representations that encompass both modalities. SPADE is trained using a large collection of Visium data from the HEST-1K dataset to generate a rich, expressive latent representation space that spans multiple tissue types (Jaume et al., 2024a). We hypothesize that training a contrastive learning model on paired ST and WSI data will create a shared latent space that captures molecularly informed features of tissue organization. This enriched representation is expected to improve performance across various downstream tasks compared to conventional WSI self-supervised learning. Recent work has explored multimodal integration of histology and spatial transcriptomics. Omni-CLIP(Loki) uses dual-encoder contrastive learning to align H&E images with transcriptomic “sentences” (W. Chen et al., 2025), while HESCAPE systematically benchmarks cross-modal contrastive pretraining strategies (Gindra et al., 2025). PathOmCLIP leverages pretrained unimodal foundation models with a set transformer to gather localized neighborhood to predict spatial gene expression (Lee et al., 2024). However, a fundamental challenge across these approaches is that training a single contrastive model on diverse tissue types can lead to trivial solutions where the model learns to distinguish tissues by type rather than capturing meaningful molecular-morphological relationships. SPADE’s architecture, based on a mixture-of-experts, is specifically designed to handle the complexities of spatially resolved paired ST and H&E data. Each expert is trained on a distinct subset of ST–WSI pairs, identified through a two-step K-means clustering process. First, patches are grouped into K subclusters within each organ, yielding per-organ centroids. Then, these centroids are clustered again across all organs to arrive at a final set of K representative clusters spanning the 515 Visium WSIs. This process generates hard negatives, enhancing training for individual experts and ensuring robust representation learning across multiple tissue types. Our contributions are summarized as follows:

1. **Multimodal Representation Learning:** We propose SPADE, a histology foundation model framework that incorporates paired H&E and ST data to enrich latent representations for pathology tasks.
2. **Mixture of Data Experts with Hard Negative Mining:** By clustering WSIs in a two-step K-means process, SPADE identifies challenging examples that enhance discrimination and prevent trivial solutions, especially across diverse organ types.
3. **Comprehensive Evaluation across 20 Tasks:** We demonstrate SPADE’s effectiveness on eight cancer classification tasks, six survival prediction tasks, and six biomarker prediction tasks, showcasing its robust performance in modeling the complexity of tumor samples.

## 2. Related work

### 2.1. Contrastive learning pretraining for multimodal datasets

Contrastive learning is a core self-supervised approach for large imaging datasets, which uses the InfoNCE loss, introduced by van den Oord et al. (2018), to pull positive pairs of data points closer together while pushing negative pairs apart. Contrastive language–image pretraining (CLIP) extends this technique to multimodal data by using paired images and captions (Radford et al., 2021). Rather than focusing solely on images, CLIP creates a multimodal embedding space using a symmetric InfoNCE loss that matches paired images and captions as positives and mixes random images and captions as negatives. This symmetric process (image-to-caption and caption-to-image) allows CLIP to develop a latent space where images and their corresponding captions cluster together.

BLEEP (Xie et al., 2024) applies the CLIP framework to spatial transcriptomics for gene expression prediction, creating a multimodal model specifically for Visium data but trained on a single tissue type, limiting its generalizability. Another model, TANGLE, recently developed by Jaume et al. (2024b) applies the CLIP framework to paired H&E WSIs and bulk RNA-seq data from the Cancer Genome Atlas (TCGA); however, bulk RNA sequencing requires tissue dissociation and lacks one-to-one matching of gene expression to tissue. SPADE addresses these limitations by training on multiple tissue types using a Mixture of Data Experts approach, utilizing the one-to-one correspondence between 20× H&E patches and their spatially resolved ST gene expression profiles to learn rich, molecularly informed patch-level embeddings for multiple tissue types. More recently, several studies have explored multimodal alignment of histology and transcriptomic data at scale. OmniCLIP (Loki) (W. Chen et al., 2025) applies CLIP-based contrastive pretraining learning by converting gene expression profiles into ‘sentences’ of ranked gene symbols, training a dual-encoder model on 2.2 million paired H&E and ST data across 32 organs. HESCAPE (Gindra et al., 2025) provides a comprehensive benchmark of cross-modal pretraining strategies for paired histology and transcriptomics, while PathOmCLIP (Lee et al., 2024) aggregates local histologic neighborhoods for spatial gene expression prediction. While these studies demonstrate the promise of multimodal alignment, they rely on a single shared encoder across highly heterogeneous tissue types, which can lead to suboptimal representations. SPADE addresses these limitations by explicitly modeling tissue heterogeneity through a Mixture of Data Experts framework. Rather than learning a single global contrastive model, SPADE partitions paired ST-WSI data into semantically coherent subsets and trains specialized experts on each subset. By leveraging the one-to-one correspondence between 20× H&E patches and their spatially resolved ST gene expression profiles, SPADE learns rich, molecularly informed patch-level embeddings that generalize across multiple tissue types.

### 2.2. Clustering for non-trivial contrastive learning

SPADE is trained on a dataset that spans multiple organs, leading to a risk of the original CLIP framework finding trivial solutions by contrasting different tissue types with each other instead of learning a meaningful latent space. Because CLIP samples negative pairings from within each batch, highly varied images in a batch can make these pairings easier for the model to learn. Ma et al. proposed a Mixture of Data Experts (MoDE) approach to address the limitations of CLIP using non-trivial negative pairings to train by incorporating a two-step K-means clustering process into training (Ma et al., 2024). MoDE first clusters semantically similar text captions, then trains a distinct CLIP model within each cluster. By sampling hard negatives from within these similar groups, the model is forced to focus on distinguishing features that differentiate similar captions and their paired images (Ma et al., 2024). Recent works have also explored clustering and prototype-guided strategies in digital pathology. For instance, Chen et al. leverage hierarchical clustering primarily for data curation and stratified batch sampling to improve the training distribution of single-modality self-supervised foundation models (B. Chen et al., 2025). Similarly, Redekop et al. uses a prototype-guided diffusion process to address data scarcity by generating synthetic pathology images (Redekop et al., 2025). While these methods demonstrate the utility of clustering in pathology, they focus on single-modality curation and synthetic generative modeling, respectively.

Using a two-step K-means procedure, we adapt this hard-negative sampling procedure to Visium data and cluster in the image feature space. In Vo et al. (2024) the authors demonstrate how hierarchical clustering can be leveraged to create high-quality representations by progressively refining clusters at different levels of abstraction. Inspired by this, our two-step K-means procedure first clusters data within each organ type, and then clusters the resulting organ-specific centroids. Our two-stage design ensures that hard negatives are sampled from semantically similar tissue contexts, forcing the model to learn fine-grained molecular distinctions rather than relying on obvious organ-level differences. This clustering approach allows our model to learn a more detailed and expressive latent space for each expert while also allowing flexibility to work across cancer types and vastly different tissue makeups.

## 3. Methods

We present the **S**patial transcriptomics and **P**athology **A**lignment using a mixture of **D**ata **E**xperts (SPADE) framework. (Fig. 1). The proposed framework acts as an image encoder, where the extracted embeddings are subsequently aggregated to generate a comprehensive slide-level representation for downstream applications. SPADE is a mixture of data experts {f(·∣c)}, each pretrained using a contrastive objective to align an H&E patch embedding with a gene expression embedding (Section 3.1) derived from a specific subset of data c as defined in Section 3.2. Following the training of data experts (Section 3.3), SPADE is comprehensively evaluated on diverse downstream tasks to validate its effectiveness (Section 4.4). Implementation details can be found in Appendix B.

### 3.1. Multimodal alignment

SPADE utilizes a contrastive learning framework inspired by CLIP, designed with a modified loss function to learn a bimodal embedding that aligns image patches with their corresponding gene expression data (Xie et al., 2024). Specifically, rather than treating gene names as text strings, our model uses raw gene expression values during training. We incorporate these raw gene expression profiles after selecting the relevant genes using Scanpy’s highly variable gene analysis, adhering to the preprocessing and methodology established in BLEEP (Xie et al., 2024). In contrast to natural images, it is common to sample multiple spots with similar gene expression profiles or image morphology within the same batch, which the contrastive objective should not pull apart. To take into account similar pairs, the similarity-adjusted target t is computed as follows:

(1) $$t=\frac{\sigma \left(\text{sim}\left(h_{x},h_{x}\right)+\text{sim}\left(h_{v},h_{v}\right)\right)}{2}\cdot \tau$$

where $h_{v}$ is an m-dimensional image embedding, $h_{x}$ is an m-dimensional gene expression embedding, and τ is a temperature parameter. The contrastive objective ℒ is then updated to align $h_{v}$ and $h_{x}$ in the latent space and is computed with cross-entropy (CE) as follows:

(2) $$ℒ=\text{mean}\left(\text{CE}\left(\text{sim}\left(h_{v},h_{x}\right),t\right)+\text{CE}\left(\text{sim}{\left(h_{v},h_{x}\right)}^{⊤},t^{⊤}\right)\right)$$

During this contrastive training process, the base image encoder is kept entirely frozen. The training is strictly limited to the expert-specific projection heads responsible for generating the final image embeddings $\left(h_{v}\right)$ and gene expression embeddings $\left(h_{x}\right)$.

### 3.2. Clustering

As shown in Fig. 2, each WSI $X_{j},j=1,\ldots ,J$ from the pretraining cohort D of size J is first divided into a set of non-overlapping patches $X_{j}=\left\{x_{j}^{1},\ldots x_{j}^{N_{j}}\right\}$, where each patch $x_{j}^{n}\in R^{W\times H\times 3}$. A pre-trained foundation model (UNI (Chen et al., 2024)) serves as the feature encoder $f_{\text{enc}}(\cdot )$ used to extract a compressed embedding from each patch. This results in a set of embeddings $H_{v,j}=\left\{h_{v,j}^{1},\ldots ,h_{v,j}^{N_{j}}\right\}$ where $h_{v,j}^{n}=f_{\text{enc}}\left(x_{j}^{n}\right)\in R^{m}$.

The goal is to extract a set of image clusters $\left\{h_{v,1},\ldots ,h_{v,C}\right\}$ with $h_{v,c}\in R^{m}$ based on the set of patch-level embeddings to formulate conditions C for data expert training. To train these experts, we utilize a two-step K-means clustering approach (see Fig. 2). In the first step, we apply fine-grained clustering utilizing patch-based embeddings within each organ type to identify clusters where the samples share similar semantics. In the second step, we aggregate the fine-grained clusters based on each cluster’s centroid to establish coarse-grained clustering, facilitating specialization among data experts across different organs.

Given the large size of the pretraining dataset 𝒟, K-means is performed on a subset of the data, as training K-means on the entire dataset may be computationally inefficient. We draw a uniform sample from each organ-specific subset (32 organs in total): ${𝒟}_{i}^{\prime }\sim {𝒟}_{i}$ and $\left|{𝒟}_{i}^{\prime }\right|\ll \left|{𝒟}_{i}\right|$, where 1≤i≤32. Then fine-grained K-means is performed over each $D_{i}$:

(3) $$S_{i}\leftarrow \text{K-means}\left({𝒟}_{i}^{\prime }\right),$$

where $S=⋃S_{i}$ - set of learned cluster centers.

A second round of clustering is performed on top of previously found cluster centers S:

(4) C←K-means(S),

where each c∈C becomes a condition for a data expert f(·∣c).

To determine the optimal number of clusters for both steps, we performed a within-cluster sum of squares (WCSS) analysis and used the elbow method to identify the point where adding more clusters provided diminishing returns. This ensured that our clustering approach captured meaningful biological variation while avoiding excessive fragmentation of the data. More details in Appendix C.

### 3.3. Data experts training

In this work, we propose to train a set of contrastive models {f(·∣c)} independently given clusters C, such that c∈C. The training data for a data expert {f(·∣c)} is:

(5) $${𝒟}_{c}=\underset{s\in S_{c}}{⋃}{𝒟}_{s},$$

where ${𝒟}_{s}=\left\{d|s={\text{argmin}}_{s\in S}\left(\|h_{v,s}-h_{v,c}\right){\|}_{2}^{2},d\in 𝒟\right\}$ is the data assigned for each fine-grained cluster with $h_{v,s}$ and $h_{v,c}$ being the image embeddings for training example s and fine-grained cluster center c respectively. $S_{c}$ is a set of fine-grained clusters assigned to each data expert f(·∣c), further trained only on $D_{c}$.

### 3.4. Slide-level representation

The proposed expert-based approach provides multiple models to select from during inference rather than relying solely on a single contrastive model. We evaluate three distinct weighting techniques for routing data experts by formulating their outputs as a weighted sum for simplicity:

(6) $$\sum_{c\in C}f(\cdot |c)\cdot p_{i}\left(c|\left(h_{v,s},h_{v,c}\right)\right),$$

where $p_{i}\left(c|\left(h_{\nu ,s}h_{\nu ,c}\right)\right)$ represents the normalized weight assigned to each expert, satisfying $\sum_{c\in C}p_{i}\left(c|\left(h_{v,s},h_{v,c}\right)\right)=1$. Here, i∈1,2,3 denotes three weighting techniques being compared, $h_{v,s}$ is the sample’s image embedding, and $h_{v,c}$ is the expert’s centroid. In the first approach, each data sample is routed to a single expert based on the proximity of its embedding to the expert centroids. Specifically:

(7) $$p_{1}\left(c|\left(h_{v,s},h_{v,c}\right)\right)=\left\{\begin{array}{ll} 1 & \text{if}\;c=\text{arg}\;{\text{min}}_{c^{\prime }\in C}\;d_{c^{\prime }}\\ 0 & \text{otherwise} \end{array}\right.$$

where $d_{c^{\prime }}=\left(\|h_{v,s}-h_{v,c^{\prime }}\right){\|}_{2}^{2}$. In the second approach, the outputs from all experts are averaged for each data sample:

(8) $$p_{2}\left(c|\left(h_{v,s},h_{v,c}\right)\right)=\frac{1}{C}$$

In the third approach, the embedding is compared to all centroids to compute distances ${\left\{d_{c}^{\prime }\right\}}_{c^{\prime }\in C}$, and the outputs are combined using a softmax-based weighting:

(9) $$p_{3}\left(c|\left(h_{v,S},h_{v,c}\right)\right)=\frac{\text{exp}\left(-d_{c^{\prime }}\right)}{\sum_{i\in C}\text{exp}\left(-d_{i}\right)}.$$

After features are obtained for each patch through one of the weighting techniques, a slide-level feature vector can be derived through a learnable aggregation method. Specifically, we employed Attention-Based Multiple Instance Learning (ABMIL) (Ilse et al., 2018), which allows for aggregating patch-level embeddings into a slide-level representation. In this setup, each patch embedding is treated as an instance, and the model learns to assign attention weights to each instance based on its relevance to the slide-level prediction task. ABMIL leverages an attention mechanism to weight each patch’s contribution, capturing spatial heterogeneity across the slide. The resulting slide-level feature vector can then be utilized for various downstream tasks, as described in Section 4.1.

## 4. Experiments and results

### 4.1. Datasets

Each dataset was processed in a similar manner with a fixed stain normalization and 224 × 224 patching at magnification 20x. The Visium gene expression data was processed by normalizing each spot to its total count, followed by log normalization. Subsequently, highly variable genes were identified using the Scanpy package (Wolf et al., 2018). The union of the top 50 most highly variable genes from each sample was used for training and prediction, resulting in 7986 genes following the method outlined in Xie et al. (2024). Additional details can be found in Appendix.

#### Pretraining

For pretraining, we utilized the HEST-1k dataset, a diverse collection of 1229 spatial transcriptomic profiles, each paired with a WSI. HEST-1k spans 153 cohorts, covering 26 organs, and includes 367 cancer samples across 25 types. For this work, we utilized only the Homo sapiens samples obtained using Visium technology, leading to a total of 515 profiles.

#### Subtyping

We evaluated the proposed approach on six different subtyping tasks: Non-Small Cell Lung Carcinoma (NSCLC) subtyping on PLCO (Zhu et al., 2013) and CPTAC (Grossman et al., 2016) (two classes), ISUP grading based on Prostate cancer grade assessment (PANDA) challenge (Bulten et al., 2022) (six classes), classification of breast cancer metastases in lymph nodes (Camelyon16) (Bejnordi et al., 2017; Litjens et al., 2018) (three classes), ovarian cancer subtypes classification (UBC-OCEAN) challenge (Farahani et al., 2022; Asadi-Aghbolaghi et al., 2024) (five classes), and PLCO Breast cancer subtyping classification (three classes) (Zhu et al., 2013). For each dataset where patient identifiers were provided, all patient WSIs were aggregated into one set of vectors for input to ABMIL.

#### Survival

We evaluated the proposed method on five different survival tasks: Breast Invasive Carcinoma (BRCA) from TCGA and PLCO, Non-Small Cell Lung Carcinoma (NSCLC) from PLCO, Uterine Corpus Endometrial Carcinoma (UCEC) from TCGA, Prostate cancer progression-free survival (PRAD) from TCGA.

#### Prostate Cancer Biochemical Recurrence

We evaluated the proposed solution on one prostate cancer biochemical recurrence dataset of whole-mount radical prostatectomies obtained from our institution.

#### Gene Biomarker Prediction

Following the dataset curation of Kather et al. (2020), we evaluated our method on six gene-alteration predictions tasks. For colorectal cancer, we combined TCGA-COAD and TCGA-READ (TCGA-CRC) to predict occurrence of driver mutations in KRAS, BRAF, and TP53. For breast cancer, we used TCGA-BRCA to predict receptor status (HER2, PR, ER).

### 4.2. Baselines and ablations

We compared our approach against five baseline vision encoders: UNI, TANGLE (pancancer-trained) (Section 2), CONCH (Lu et al., 2024) Prov-GigaPath (Xu et al., 2024), and Loki (W. Chen et al., 2025). For the classification tasks, (specifically subtyping and gene prediction), both TANGLE and Prov-Gigapath were evaluated using a linear probe setup. Because TANGLE provides a direct slide-level vector representation, we assessed its slide-level features by following the linear probe framework and codebase established in their original study. Similarly, Prov-Gigapath provides a slide-encoder model that consolidates patch-level embeddings into a single feature vector, which we subsequently used to fit a linear probe for the classification targets. Conversely, for the survival tasks, rather than using a linear probe, we used a lightweight multi-layer perceptron (MLP) trained with the same survival negative log-likelihood loss that is used to train the patch-level feature ABMIL models.

Alongside the baseline comparisons, we conducted an ablation study to evaluate different strategies for routing data experts (Sections 3.4, 4.4.4). We further examined the impact of varying the number of clusters in both stages of the two-step clustering procedure (Section 4.4.4), as well as a simplified variant of SPADE using one-step K-means clustering to assess the contribution of the additional clustering step (see Appendix E).

To thoroughly evaluate SPADE, we conducted a comparison with a single-expert model (SPADE-0) across subtyping, survival, and BCR prediction tasks (see Appendix F).

In addition, we explored the effect of the underlying foundation model by replacing the UNI encoder with the Virchow2 foundation model (Zimmermann et al., 2024) within SPADE and compared its performance to both standard Virchow2 features and a single-expert version of SPADE trained under the same settings (see Appendix G).

### 4.3. Implementation details

We used publicly available implementations and pre-trained weights for all baseline foundational models in our study. The preprocessing pipelines were adopted from the official repositories of each model, ensuring consistency with their original training procedures. No additional fine-tuning or retraining was performed on these models beyond their publicly released checkpoints.

To ensure rigorous evaluation and prevent data leakage, all datasets were partitioned the patient level into a label-stratified train/validation/test split of 70/10/20. The 20% set was strictly held out for final evaluation. Using the remaining 80% of data, we performed a systematic hyperparameter search using Optuna with 5-fold cross-validation. The optimal hyperparameters for our model and the applicable baselines were selected based on this cross-validation performance. Once the optimal configurations were identified, the final models were fit and evaluated on the aforementioned 70/10/20 split.

Specifically we trained the classification ABMIL from scratch for each task using 5-fold cross-validation hyperparameter tuning to select the best hyperparameters for each task based on cross-fold validation performance (see Appendix B). For the subtyping and gene prediction classification tasks, Prov-Gigapath and TANGLE did not undergo Optuna hyperparameter tuning, as these representations were fit using simple linear probe models. Conversely, for the survival tasks, both Prov-Gigapath and TANGLE underwent Optuna-based hyperparameter tuning to optimize the parameters of their respective lightweight MLP heads.

### 4.4. Results

#### 4.4.1. Cancer subtyping

Evaluated under the rigorous cross-validation setup, SPADE demonstrates distinct advantages in cancer subtyping tasks. Notably, SPADE’s advantage is most pronounced in the F1 score rather than the AUC, suggesting that our model yields more balanced predictions and that SPADE’s spatially informed representations yield more discriminative features at decision boundaries. Overall, SPADE outperforms or ties all baseline models on five of the eight downstream subtyping tasks in terms of F1 score (Table 1). For instance, SPADE achieves the highest F1 scores on the PLCO-Lung, CPTAC Lung, TCGA-PRAD, UBC-Ocean and TCGA-BRCA datasets. While single modality foundation models occasionally edge out SPADE in AUC for specific datasets (e.g. CONCH on PLCO-Breast or UNI on PANDA), SPADE remains highly robust across diverse cancer tasks.

The comparison with TANGLE’s performance highlights the distinct advantage of spatial transcriptomics over Bulk RNA-seq. Using Bulk RNA-seq may limit model expressibility, likely due to the loss of heterogeneity and the lack of one-to-one alignment between expression and imaging data.

#### 4.4.2. Survival prediction and prostate BCR analysis

We used the concordance index (c-index) for survival and prostate cancer biochemical recurrence (BCR) evaluation (Table 2). To overcome the limitations of overall survival, which includes deaths unrelated to cancer, we utilize disease-specific survival (DSS) for the four survival tasks (Liu et al., 2018).

Evaluated across these six datasets, SPADE maintains strong prognostic capabilities against various baselines. In direct head-to-head comparison, SPADE outperforms UNI on four out of six tasks, CONCH on five out of six tasks, and GigaPath on three out of six tasks. SPADE notably secures the absolute highest c-index on the TCGA-BRCA and PLCO-Breast cohorts. Furthermore, SPADE consistently outperforms TANGLE across all datasets, demonstrating the critical importance of spatial context.

#### 4.4.3. Gene biomarker prediction

Predicting gene alterations and receptor status from histology slides alone is challenging, and the datasets often suffer from high imbalance. Despite this, SPADE secures the highest AUROC on three out of the six tasks evaluated (ER, PR, KRAS), often by large margins (Table 3). In head-to-head comparisons, SPADE demonstrates strong superiority over individual baselines. SPADE outperforms UNI on four of the six tasks in AUC and three in F1, and similarly outperforms CONCH on four of the six tasks for both AUC and F1.

#### 4.4.4. Ablation studies

For the data experts’ routing ablation studies, there is varying performance between different routing techniques (Table 4, 5). For most subtyping datasets, $p_{2}$ (averaging) and $p_{3}$ (distance weighting) routing methods perform better than $p_{1}$, which simply uses the expert of the closest centroid. This suggests that incorporating additional model input from other experts through ensembling provides a performance advantage, potentially enabling more complex representations of the WSI.

Our ablation study on the two-step clustering procedure revealed that varying the number of clusters at each stage ($k_{1}$ and $k_{2}$) can influence model performance, but the improvement varies across different datasets and cancer types (see Table 6, 7). For the subtyping task, the configuration with $k_{1}=16,k_{2}=16$ achieved the highest AUC in six datasets, demonstrating the effectiveness of balanced hierarchical clustering. Increasing the second-stage clustering granularity $\left(k_{2}=32\right)$ improved AUC and F1 scores in one case, such as Camelyon16, suggesting that a finer-grained latent space can enhance classification precision, but it also requires double the computational resources or double the runtime. Reducing the second-stage clustering granularity $\left(k_{2}=8\right)$ led to mixed results: while it improved F1 in some datasets, it generally resulted in lower AUC scores.

For the survival and BCR, $k_{1}=16,k_{2}=16$ achieved the highest c-index for two out of six tasks, demonstrating the effectiveness of balanced clustering. Increasing $k_{1}$ and $k_{2}$ to 32 improved the c-index for TCGA-PRAD while reducing $k_{2}$ to 8 led to performance drops across most survival tasks.

Further comparisons between SPADE and its single-expert version highlight the significant impact of the mixture of data experts module on the performance, as evidenced by SPADE’s consistently higher AUC and F1 score (see Appendix E). This trend may be attributed to the diverse tissue types represented in the HEST-1k dataset (e.g., brain, heart, lung). A model trained on all organ types might lack the specificity needed to improve performance for each task. Given the high variability of genes present between tissue types, training on data from all organs within a single model may limit its ability to capture the heterogeneity essential for effective subtyping and survival prediction. Additionally, as discussed in Section 2.2, with such diverse tissue types, contrastive learning could rely on easy negative pairings to drive down the loss, resulting in a less expressive latent space.

#### 4.4.5. Interpretability analysis

We conducted our first interpretability analysis by plotting the attention weights from the ABMIL variants on our in-house UCLA Prostate BCR dataset to better understand the differences between the SPADE and UNI features. In Fig. 3, we present attention heatmaps, where deeper red indicates higher attention weights, and the predicted cancer mask is shown in yellow. We validated this cancer prediction mask against pathology reports. In panel (a), UNI attention highlights some small regions within the tumor boundary, but much of the attention is diffuse. In contrast, the SPADE framework’s attention weights display higher attention regions within the cancer region and a hot zone along the bottom right border of the tumor mask.

The heatmap depicts which regions of the WSI had the highest attention for a case from the UCLA BCR task. The attention heatmap produced by SPADE highlights areas predominantly showing poorly formed and compact 4 + 5 and 4 + 3 tumor patterns with tertiary 5 according to the Gleason grading system (Epstein et al., 2016), subtle desmoplastic stromal response with slightly cellular fibroblasts exhibiting pale cytoplasm and nuclear enlargement.

#### 4.4.6. Comparison of TCGA-CRC attention heatmaps from SPADE vs. UNI

To compare SPADE and UNI for gene-related tasks, attention weights from the TCGA-CRC BRAF mutation task were extracted to identify differences in the attentions of each model.

We created a series of heatmaps similar to our setup for Prostate BCR attention visualization (see Fig. 4, D.8, D.9). Additionally, we extracted these heatmaps at high resolution and created a setup for a board-certified pathologist to overlay the heatmap on tissue at full resolution. This setup allowed the pathologist to zoom in on “hot” regions and examine the underlying tissue patterns. From the heatmaps, the pathologist observed that UNI tended to focus on non-neoplastic areas containing smooth muscle and chronic inflammation, rather than tumor glands. In contrast, the pathologist noted that SPADE focused on areas with high grade dysplasia and invasive carcinoma. In one case SPADE also focused on regions with elongated nuclei (“spindle”-like). While spindle-cell morphology is not a canonical pattern of BRAF in colorectal cancer samples, spindle-cell patterns have been described in non-CRC BRAF-altered neoplasms such as melanoma (Suurmeijer et al., 2018). In another case, the pathologist noted that UNI’s attention pattern appeared more random, focusing on necrotic regions rather than following the glandular architecture. Overall, in the six cases reviewed by our pathologist, UNI focused primarily on smooth muscle tissue and regions without tumor, whereas SPADE concentrated more on tumor regions.

Next, we extracted the 50-highest attention patches from five different cases at 20x magnification for both SPADE and UNI (see Fig. 5). We then provided patches to the pathologist, grouped by model and case, with model names obscured. Across the five cases, SPADE generally highlighted malignant glands, often with high-grade or cribriform architecture. UNI, in contrast, frequently highlighted lower-grade or fewer glands in its top 50 highest attention patches, and often emphasized stroma, fibrotic tissue, or inflammation rather than the tumor. In several cases, UNI identifies tiles with little or no carcinoma. The clinical impression suggests that SPADE is more tumor-focused, while UNI shifts attention to stromal or non-cancerous regions.

## 5. Discussion

Our study demonstrates that training on paired spatial transcriptomics and histology data substantially enhances the expressiveness of deep histology features across diverse cancer tasks. To rigorously validate this, we evaluated SPADE under a strict 5-fold cross-validation framework across 20 downstream tasks, including subtyping, gene prediction, and survival analysis. Under this setup, SPADE consistently achieved state-of-the-art results and demonstrated a significant advantage in producing highly balanced predictions. Specifically, SPADE’s spatial transcriptomics-informed representations provided superior F1 scores on the majority of the classification tasks when compared head-to-head against leading single-modality and multi-modality baselines such as UNI, CONCH, and Prov-Gigapath. These results highlight the value of multimodal supervision in building more robust histopathology foundation models. As a complementary analysis, we evaluated SPADE on the HEST-1K gene expression prediction benchmark after excluding benchmark samples from pretraining and retraining on the updated split (Supplementary Appendix J). SPADE expert-only embeddings showed lower performance than UNI features, likely because the low-dimensional contrastive expert projection is not optimized for linear gene-expression reconstruction. However, SPADE+UNI features performed comparably to UNI alone, while SPADE’s primary gains were observed in downstream classification and survival tasks.

Our method introduces a two-step clustering procedure coupled with a mixture-of-data-experts contrastive learning approach, enabling effective hard-negative mining for multi-organ histopathology representation learning. Crucially, the core technical novelty of SPADE lies in using this MoDE architecture to solve a data bottleneck in contrastive alignment during CLIP training. When projecting highly heterogeneous H&E images and spatial transcriptomics data into a shared latent space without routing, models can easily minimize the contrastive loss by recognizing broad, obvious morphological differences between organs. SPADE’s hierarchical clustering forces the model into hard-negative mining, requiring it to differentiate between subtly different tissues within the same expert cluster. This mechanism helps the model learn fine-grained, localized features. While recent applications of clustering in computational pathology have focused on single-modality data curation and batch sampling (B. Chen et al., 2025) or synthetic data generation (Redekop et al., 2025), SPADE applies MoDE directly to multimodal representation learning on real data. Furthermore, SPADE is not merely a training technique, the framework uses these learned expert projections to perform weighted feature aggregation during inference, proving its necessity over standard clustering or simple centroid reweighting. Importantly, this approach can be extended to any histological dataset with multiple diseases and multiple organs to improve contrastive pretraining.

While we trained on the largest and most diverse public Visium dataset available, the sample size of 515 remains small compared to large digital pathology datasets. However, given the cost of acquiring spatial transcriptomics data and the relative novelty of the technique, it may be some time before datasets of comparable size to those of digital pathology can be assembled. Moreover, spatial transcriptomics gene expression data often suffer from high dropout rates, with many genes exhibiting zero expression levels and other noise-related issues. Although we attempted to mitigate this issue by clustering in the image space and using it to supervise our data experts, incorporating additional supervision using the gene expression space could enhance model robustness and leverage this data type even more.

Recent advances in spatial transcriptomics foundation models provide an opportunity to utilize pretrained representations on the gene expression side rather than relying solely on raw expression profiles. These models learn biologically meaningful latent spaces from large-scale spatial transcriptomics datasets, capturing both spatial context and transcriptional co-expression patterns that could complement histology-derived features. Integrating such pretrained embeddings (e.g., stFormer (Cao et al., 2024), or SToFM (Zhao et al., 2025)) into multimodal pretraining could further improve alignment between morphological and molecular modalities.

HEST-1k contains various types of spatial transcriptomics data, spanning in-situ imaging techniques (e.g., Xenium) and Next-Generation sequencing techniques (e.g., Visium). In our study, we chose to use only the Visium data, as it provides the whole-genome transcriptome, enabling us to identify sets of highly variable genes shared across tissue types without being restricted by organ-specific panels. In future work, we plan to incorporate other spatial transcriptomics datasets and further expand our dataset. Additionally, the 2-step K-means clustering procedure is sensitive to the choice of K, which may vary in optimality across downstream tasks. We optimized our choice of K using the within-cluster sum of squares, but additional clustering optimization with adaptive or hierarchical clustering methods could further enhance performance.

The SPADE model weights and code are publicly released to facilitate open-source use (https://github.com/uclabair/SPADE). Researchers can apply our pretrained model to their own histology datasets to obtain transcriptomics-enhanced multimodal representations.

## 6. Conclusions

Based on the performance across our 20 downstream tasks, the SPADE framework has been shown to be an expressive feature encoder using spatial transcriptomics data. Utilization of HEST-1k allowed our model to learn robust, biologically relevant embeddings by aligning histological image features with spatially resolved gene expression patterns, which set a strong foundation for downstream pathology analysis tasks. Rather than simply combining existing algorithms, SPADE represents a novel framework that successfully distills complex spatial transcriptomics data into useful histology representations for diverse tasks. Our improved performance over the single expert version of our framework highlights the benefit of SPADE’s mixture of experts approach. Additionally, our results show significant improvement over TANGLE, which relies on bulk sequencing data, underscoring the value of spatial gene profiling. SPADE’s ability to utilize spatial context allows our model to capture localized differences in gene expression that are crucial for accurate disease subtyping and survival prediction.

## Figures and Tables

**Fig. 1.**
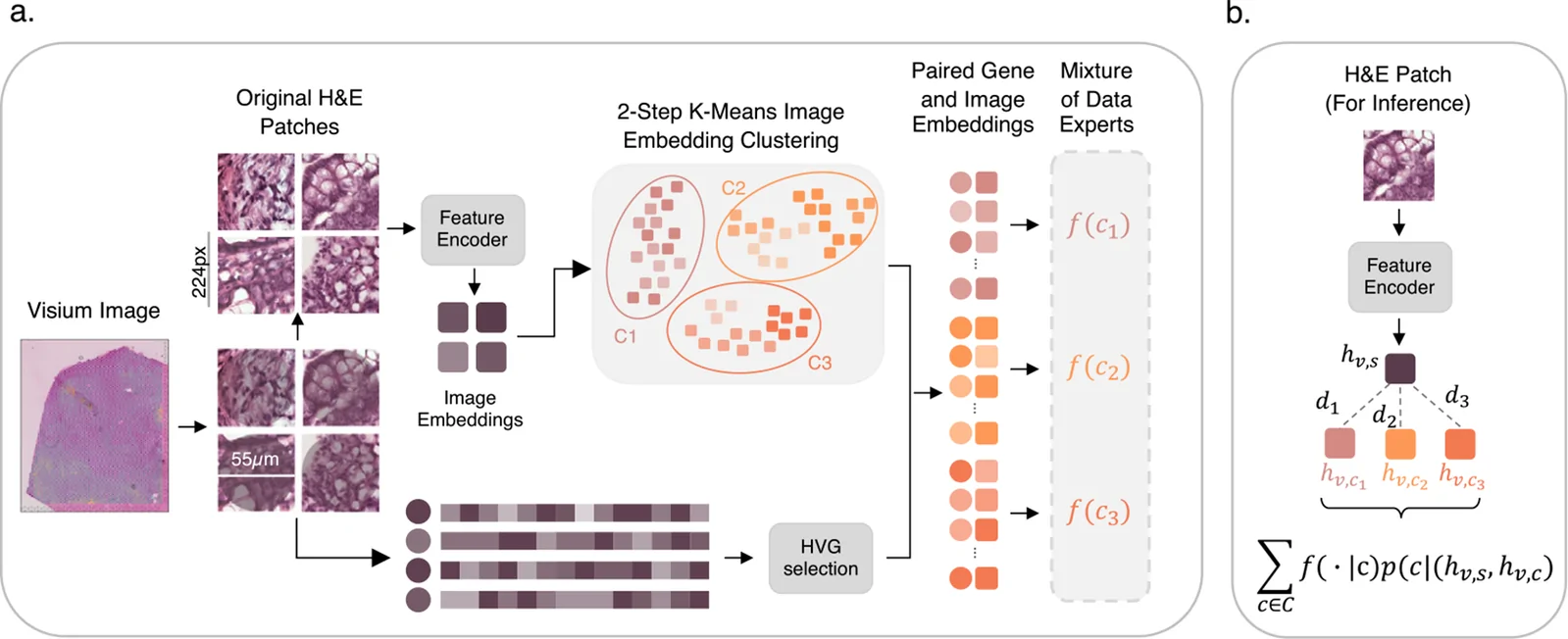
Overview of SPADE workflow. **a. A** WSI is segmented and patched into a set of non-overlapping patches. A compressed feature for each patch is obtained through a pre-trained feature encoder. A corresponding gene expression vector is obtained after preprocessing. 2-step K-Means clustering is performed to create data experts across all WSIs in the dataset. **b.** For inference, three data experts’ routing techniques are evaluated (Section 3.4).

**Fig. 2.**
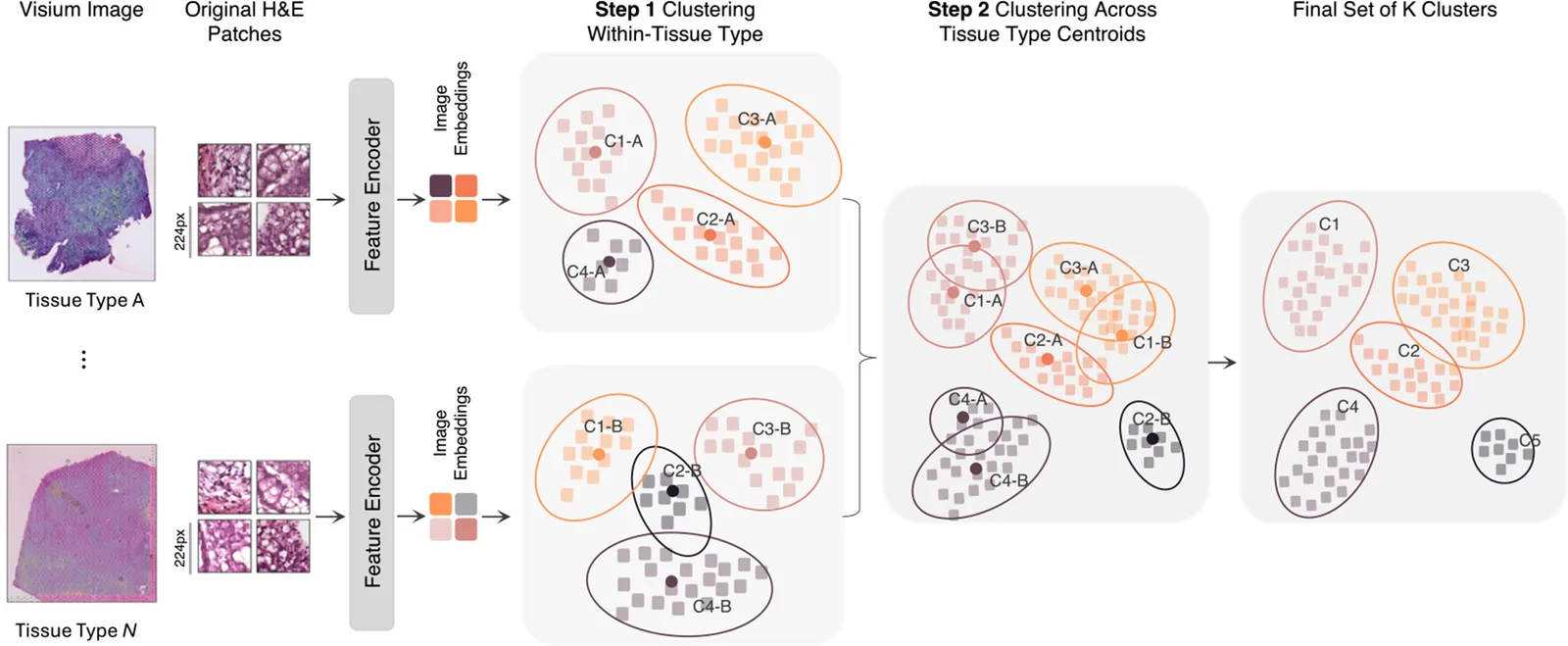
Two-step clustering framework for data expert construction. Each WSI is divided into patches and encoded into patch-level features. **Step 1:** Within each tissue type, K-Means clustering is applied to obtain tissue-specific centroids. **Step 2:** The centroids across tissue types are clustered again to form a final unified set of K clusters (data experts) used for data experts training.

**Fig. 3.**
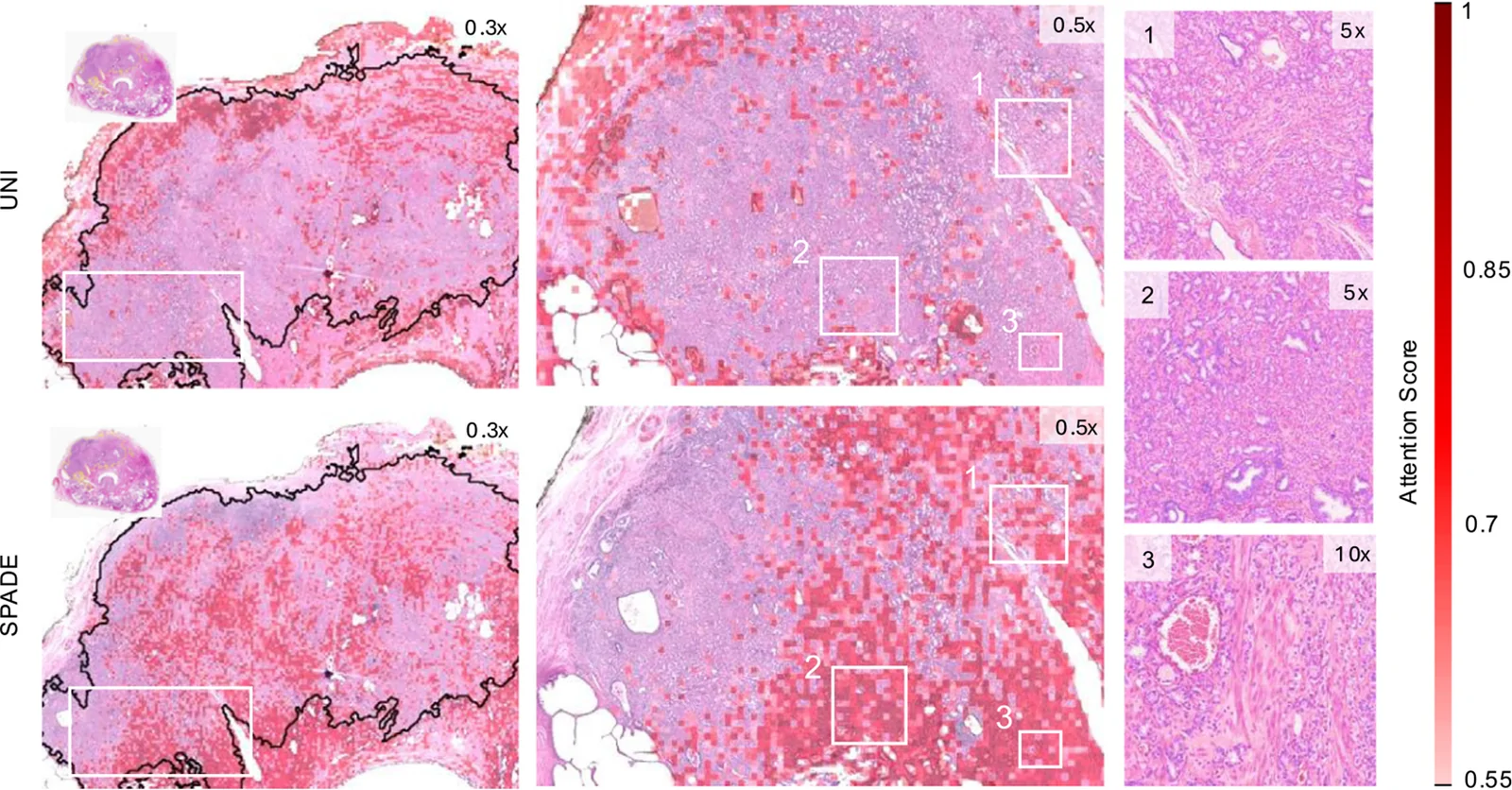
Interpretability of SPADE. Attention scores for a prostate WSI positive for biochemical recurrence (BCR) are visualized as a heatmap for the UNI and SPADE models. The deeper the red color, the higher attention the model puts on that region of the tissue. The black outline shows the predicted tumor boundary from our internal cancer prediction model for whole-mount prostectomy WSIs. SPADE shows a higher concentration of attention within the tumor boundary compared to UNI, with some areas of focus along the tumor border. Sub-crops are selected to show the patterns with high attention at higher magnifications.

**Fig. 4.**
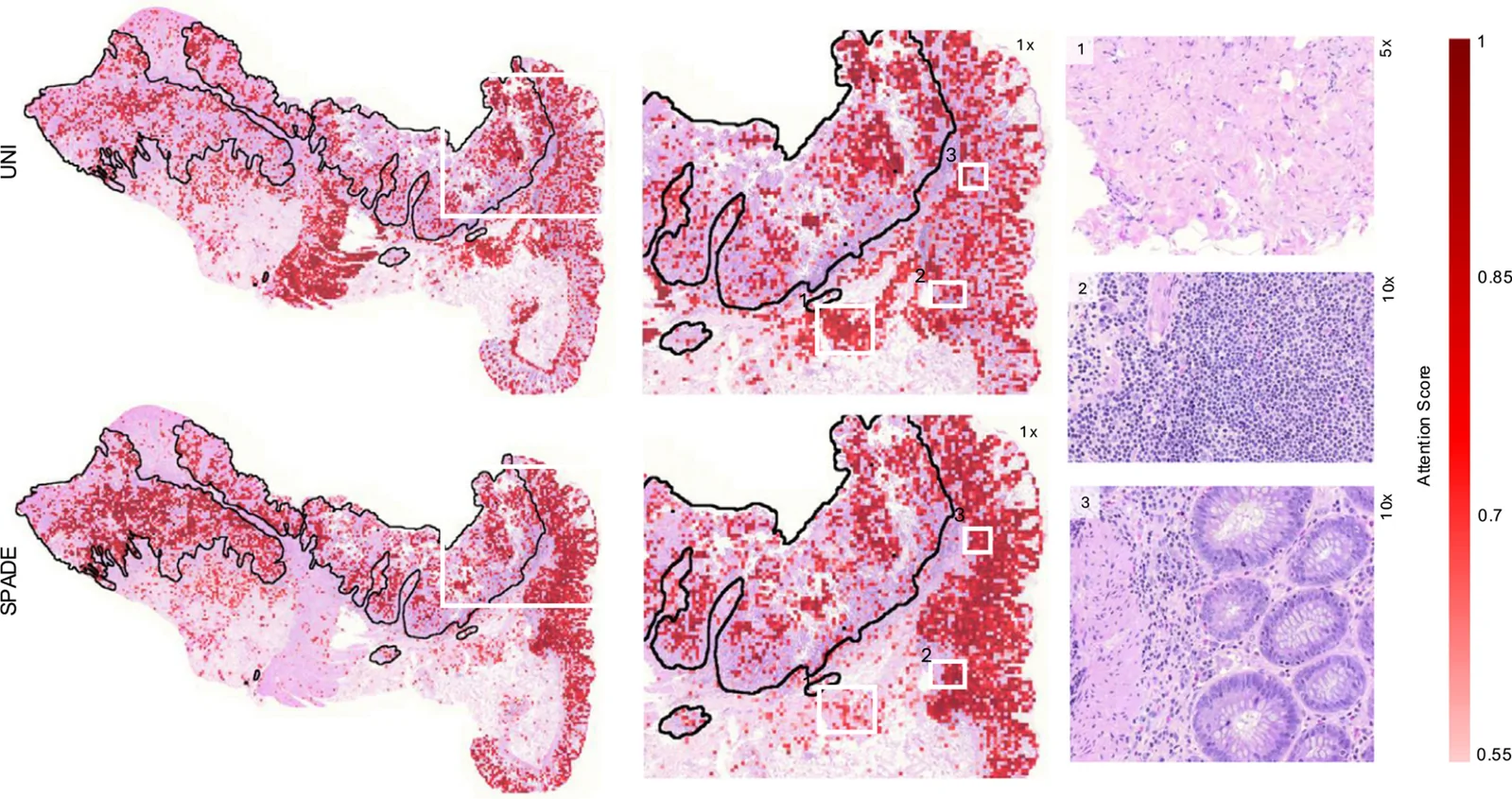
Interpretability of SPADE on TCGA-CRC BRAF Task. Attention scores for a colorectal cancer WSI are visualized as a heatmap for the UNI and SPADE models. The deeper the red color, the higher attention the model puts on that region of the tissue. Sub-crops are selected to show the patterns with high attention at higher magnifications. A pathologist noted differences between UNI and SPADE heatmaps (see Section 4.4.6). Cancer outline delineated by a pathologist in black.

**Fig. 5.**
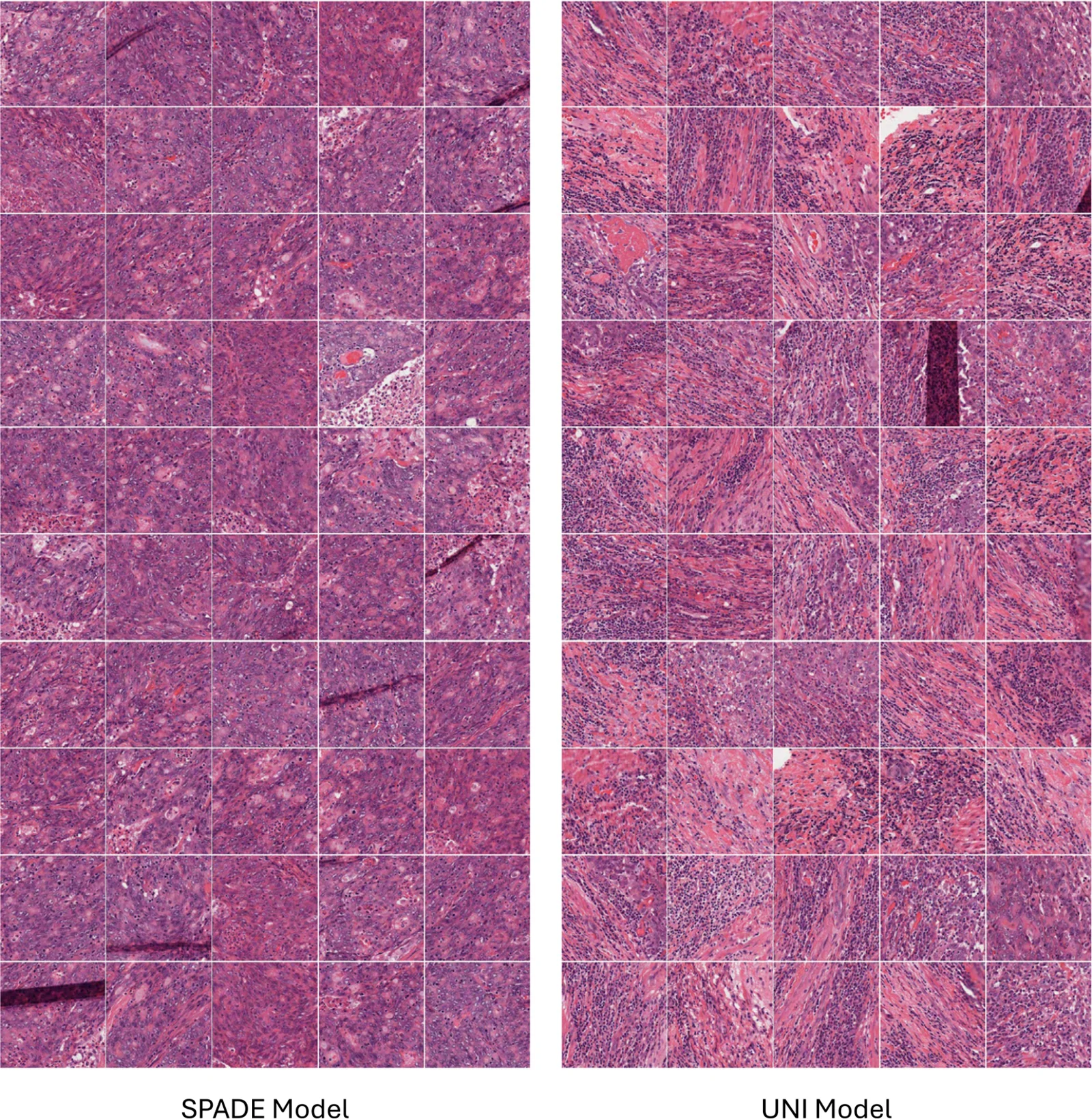
Highest Attention Patches SPADE vs UNI. We used the attention scores to extract the 50 highest-scoring attention patches from our model, SPADE, and compared them to the 50 highest-scoring attention patches from UNI. We then provided the resulting plot to a pathologist for review, with model names withheld. The pathologist noted differences between UNI and SPADE highest attention patches (see Section 4.4.6).

**Table 1:** **Subtyping prediction** results of SPADE and baselines for eight different subtyping tasks. Metrics AUC and F1 are shown as percentages. The best performance is in bold, and the second best is underlined. TANGLE could not be applied to TCGA datasets since they were used to train the model.

	Lung				Prostate				Lymph nodes		Ovarian		Breast			
	PLCO		CPTAC		PANDA		TCGA-PRAD		CAM16		UBC-Ocean		PLCO		TCGA-BRCA	
	AUC	F1	AUC	F1	AUC	F1	AUC	F1	AUC	F1	AUC	F1	AUC	F1	AUC	F1
TANGLE	95.6	91.1	96.7	89.7	88.1	57.4	–	–	74.2	57.9	96.4	81.5	76.4	55.3	–	–
Gigapath	97	90.1	97.1	89.9	72.5	33.9	75.3	38.7	93	**88.3**	94.2	77.9	69.6	50.4	69.4	63.9
UNI + ABMIL	97.1	**93.3**	**97.3**	90.9	**94.6**	**76.3**	**82.4**	44.8	**96.8**	81.4	**98.1**	87.4	78.1	55.9	96.6	96.2
CONCH + ABMIL	**97.6**	90	96.4	90.5	91.3	64.2	79.2	45.1	96.2	84.8	96.9	80.7	**82.3**	**60.8**	97	**96.6**
LOKI + ABMIL	64.2	54.6	91.4	84.3	84.3	49.9	63.5	26.2	52.7	32.7	86.8	60.2	52.9	23.8	82.6	89.2
SPADE + ABMIL	96.9	**93.3**	97.2	**91.1**	94.2	73.5	80.5	**48.8**	95.8	85.3	97.9	**89.2**	80.8	60.6	**97.3**	**96.6**

**Table 2:** **Survival and BCR prediction** results of SPADE and baselines for six different survival tasks. c-index values are shown as percentages. The best performance is in bold, and the second best is underlined. TANGLE could not be applied to TCGA datasets since they were used to train the model.

	Prostate		Lung	Uterine	Breast	
	UCLA BCR	TCGA-PRAD	PLCO	TCGA-UCEC	TCGA-BRCA	PLCO
	c-index	c-index	c-index	c-index	c-index	c-index
TANGLE	66.4	–	48.6	–	–	51.3
Gigapath	47.9	**62.6**	67.6	67.2	61.2	51.2
UNI + ABMIL	**67.9**	58.1	39.9	67.4	71.1	53.3
CONCH + ABMIL	59.9	56.3	43.4	**69.5**	65	53.2
Loki + ABMIL	67.4	55.8	**71.1**	64.4	61.2	54
SPADE + ABMIL	65.6	56.6	52.6	69.1	**71.4**	**54.5**

**Table 3:** Molecular biomarker prediction results on TCGA cohorts. Comparison of SPADE with baseline methods for predicting receptor status in TCGA-BRCA (HER2, ER, PR) and mutational status in TCGA-CRC (KRAS, BRAF, TP53). Results are reported in terms of AUC and F1 score (%). The best performance is in bold, and the second best is underlined. TANGLE could not be applied to TCGA datasets since they were used to train the model.

	TCGA-BRCA						TCGA-CRC					
	HER2		ER		PR		KRAS		BRAF		TP53	
	AUC	F1	AUC	F1	AUC	F1	AUC	F1	AUC	F1	AUC	F1
TANGLE	–	–	–	–	–	–	–	–	–	–	–	–
Gigapath	**73.3**	26.2	89.9	**93.09**	81.3	83.27	59.6	**51.6**	73.1	14.7	81	**84**
UNI + ABMIL	68.7	**37.3**	86.7	89.8	84.6	84.7	71.1	28.6	66.1	30.8	**81.5**	73
CONCH + ABMIL	68.8	40	86.7	78.5	84.1	83.2	66.4	20	**81.9**	**34.5**	75.3	73
Loki + ABMIL	60.8	35.1	76.8	81.4	61.2	80.4	48.2	10.5	64.4	25	66.5	70.8
SPADE + ABMIL	67	35.6	**92.5**	88.9	**86.3**	**88.2**	**74**	34.5	73.6	28.6	80	73.4

**Table 4:** Ablation study of the data experts’ routing method for subtyping. $p_{1}$ corresponds to the case where the expert associated with the closest centroid is selected for each data point. $p_{2}$ corresponds to the case where the outputs of all experts are averaged to produce a final prediction. $p_{3}$ corresponds to the case where the expert selection is inversely proportional to the distance (Section 3.4). Metrics AUC and F1 are shown as percentages. The best performance is in bold, and the second best is underlined.

	Lung				Prostate				Lymph nodes		Ovarian		Breast			
	PLCO		CPTAC		PANDA		TCGA-PRAD		CAM16		UBC-Ocean		PLCO		TCGA-BRCA	
	AUC	F1	AUC	F1	AUC F1		AUC	F1	AUC	F1	AUC	F1	AUC	F1	AUC	F1
$p_{1}$	**97.3**	81.1	96.9	74.6	**94.2**	**74.5**	72.7	38.9	91.3	83.3	96.9	82.7	77.4	57.9	97.5	**96.6**
$p_{2}$	94.2	92.7	96.5	78.3	94.1	73.8	80.4	**49.5**	91.8	83.4	96.8	83.9	77.4	54.8	**97.7**	96.5
$p_{3}$	96.9	**93.3**	**97.2**	**91.1**	**94.2**	73.5	**80.5**	48.8	**95.8**	**85.3**	**97.9**	**89.2**	**80.8**	**60.6**	97.3	**96.6**

**Table 5:** The ablation study of the data experts’ routing method for survival and BCR. $p_{1}$ corresponds to the case where the expert associated with the closest centroid is selected for each data point. $p_{2}$ corresponds to the case where the outputs of all experts are averaged to produce a final prediction. $p_{3}$ corresponds to the case where the expert selection is inversely proportional to the distance (Section 3.4). The best performance is in bold, and the second best is underlined. c-index values are shown as percentages.

	Prostate		Lung	Uterine	Breast	
	UCLA BCR	TCGA-PRAD	PLCO	TCGA-UCEC	TCGA-BRCA	PLCO
	c-index	c-index	c-index	c-index	c-index	c-index
$p_{1}$	63	56.2	43.9	70.4	69.4	46.9
$p_{2}$	**67**	**57.4**	50.9	**70.7**	64.4	51.7
$p_{3}$	65.6	56.6	**52.6**	69.1	**71.4**	**54.5**

**Table 6:** Ablation study of the number of experts for each clustering step for subtyping. Metrics AUC and F1 are shown as percentages. The best performance is in bold, and the second best is underlined.

	Lung				Prostate				Lymph nodes		Ovarian		Breast			
	PLCO		CPTAC		PANDA		TCGA-PRAD		CAM16		UBC-Ocean		PLCO		TCGA-BRCA	
	AUC	F1	AUC	F1	AUC	F1	AUC	F1	AUC	F1	AUC	F1	AUC	F1	AUC	F1
$16(k_{1}),16(k_{2})$	**96.9**	93.3	**97.2**	**91.1**	94.2	73.5	**80.5**	**48.8**	95.8	85.3	**97.9**	**89.2**	**80.8**	60.6	**97.3**	**96.6**
$16(k_{1}),8(k_{2})$	96.6	**95.5**	95.8	68.8	94	**73.8**	73.9	39.9	93.5	77.3	97.5	83	75.5	**60.8**	95.8	94.9
$16(k_{1}),32(k_{2})$	95	88.4	96.8	80	**94.4**	71.9	79.4	46.2	93	87.1	96.7	82.6	50	28.7	92.5	94.4
$32(k_{1}),32(k_{2})$	96.7	93.3	93.4	75.4	93.8	73	77.7	46.2	**98.3**	**88.8**	97	80.6	50.4	28.7	95.5	96.4

**Table 7:** Ablation study of the number of experts for each clustering step for survival and BCR. c-index values are shown as percentages. The best performance is in bold, and the second best is underlined.

	Prostate		Lung	Uterine	Breast	
	UCLA BCR	TCGA-PRAD	PLCO	TCGA-UCEC	TCGA-BRCA	PLCO
	c-index	c-index	c-index	c-index	c-index	c-index
$16(k_{1}),16(k_{2})$	65.6	56.6	**52.6**	69.1	**71.4**	54.5
$16(k_{1}),8(k_{2})$	63.9	56.2	45.1	70.6	68.6	**55.9**
$16(k_{1}),32(k_{2})$	**67.4**	56.1	50.3	**70.9**	71.1	50
$32(k_{1}),32(k_{2})$	66.2	**57.2**	41	69.6	69	51

**Fig. C.6.**
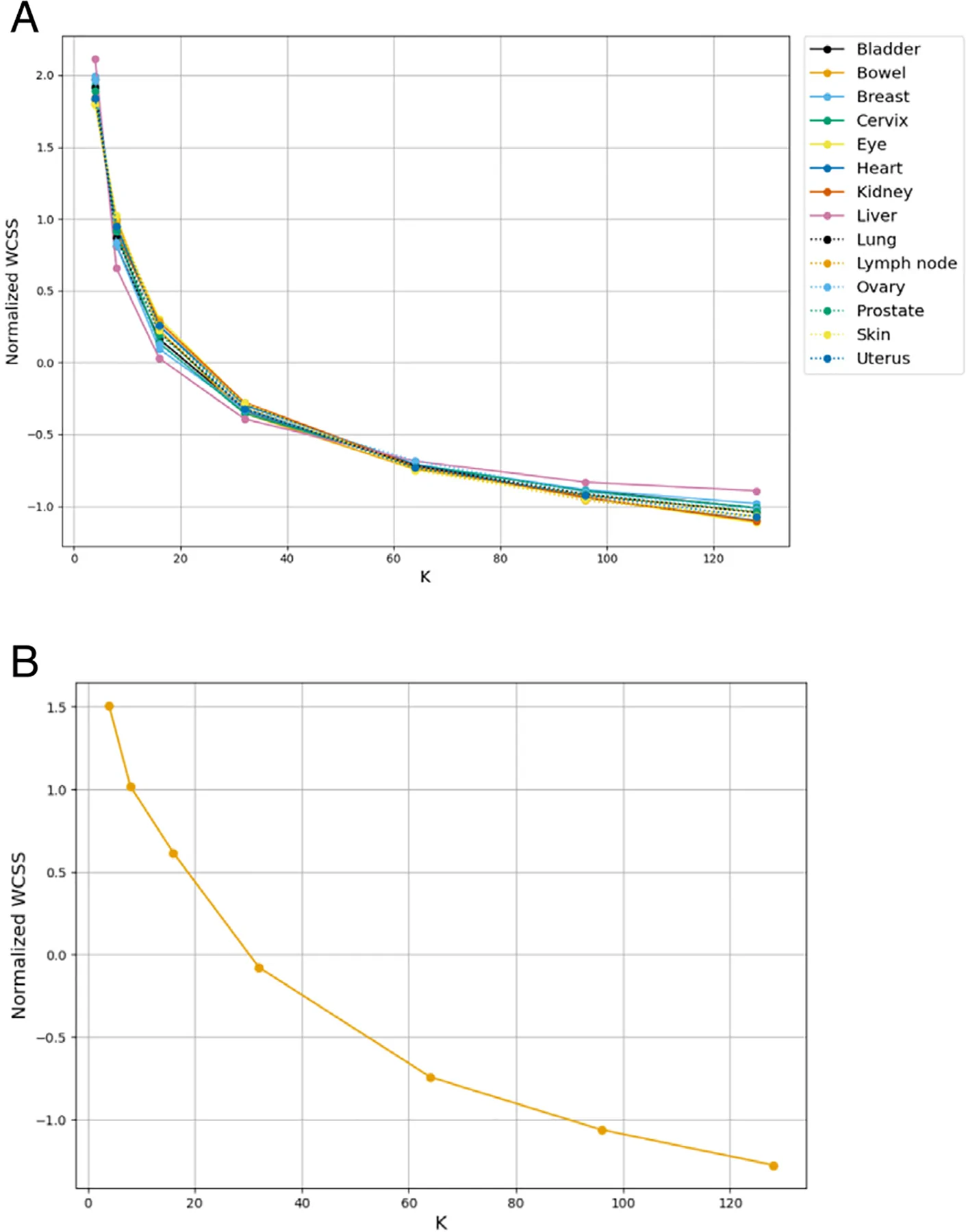
Within-Cluster Sum of Squares (WCSS) Elbow Plots Across Values of K **A.** First-level clustering performance evaluated using normalized WCSS across 14 organs. **B.** Second-level clustering performance evaluated using normalized WCSS.

**Fig. C.7.**
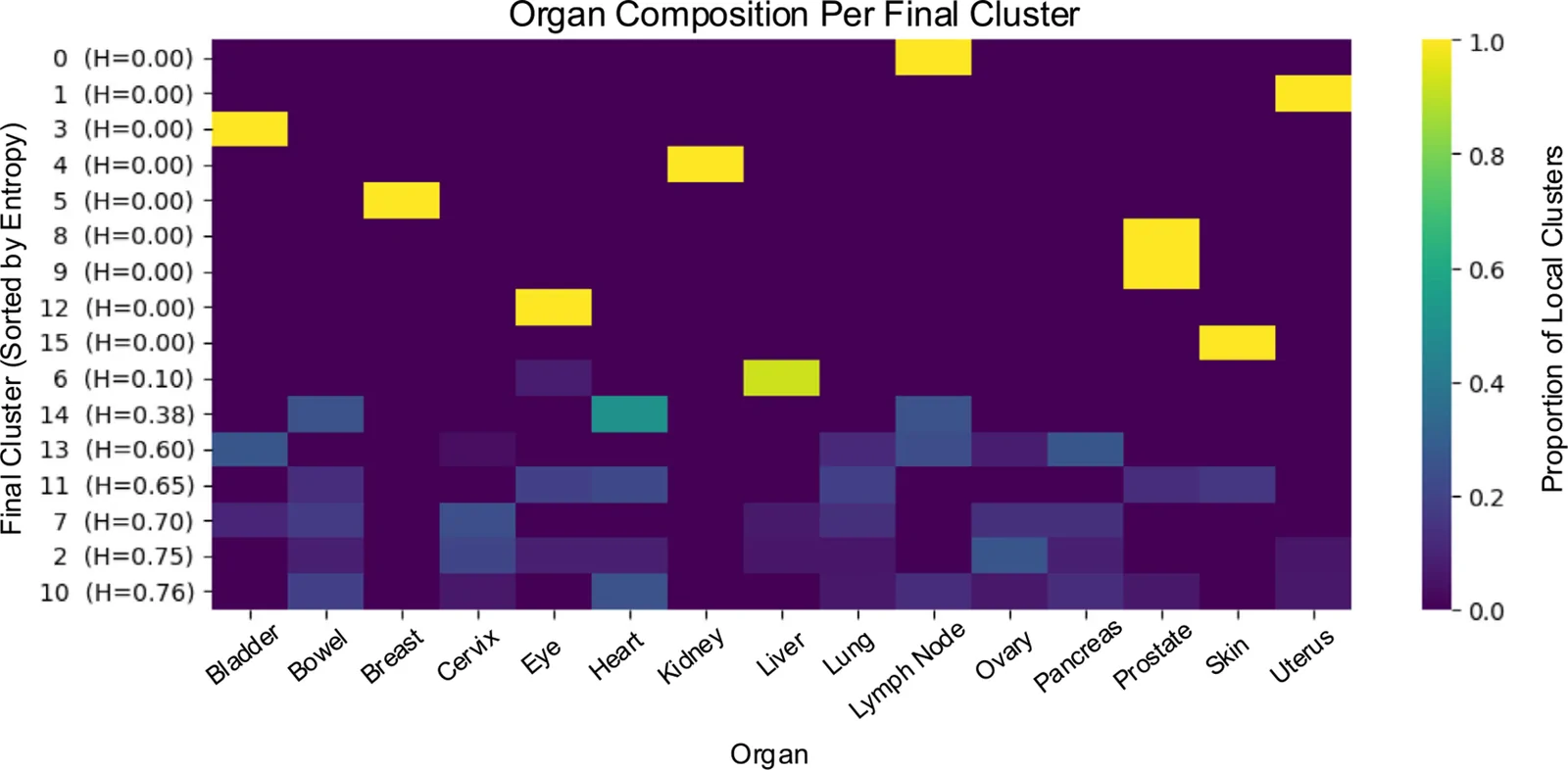
Heatmap of organ composition per final cluster. Rows capture final clusters sorted by entropy, columns represent organ, color = $p_{j,O}$.

**Fig. D.8.**
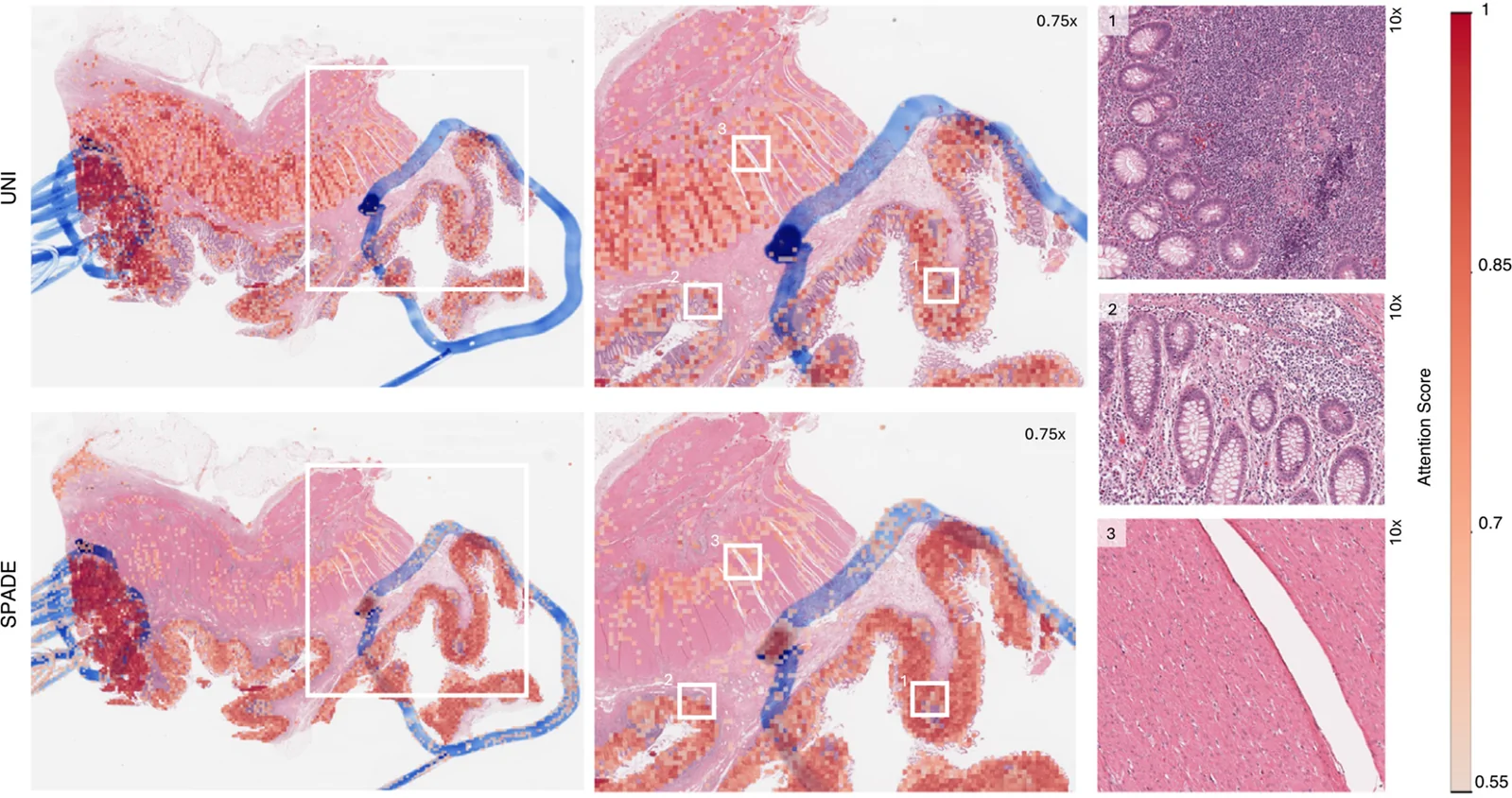
Interpretability of SPADE. Attention scores for a colorectal cancer WSI are visualized as a heatmap for the UNI and SPADE models. The deeper the red color, the higher attention the model puts on that region of the tissue. Sub-crops are selected to show the patterns with high attention at higher magnifications. A pathologist noted differences between UNI and SPADE heatmaps (see Section 4.4.6).

**Fig. D.9.**
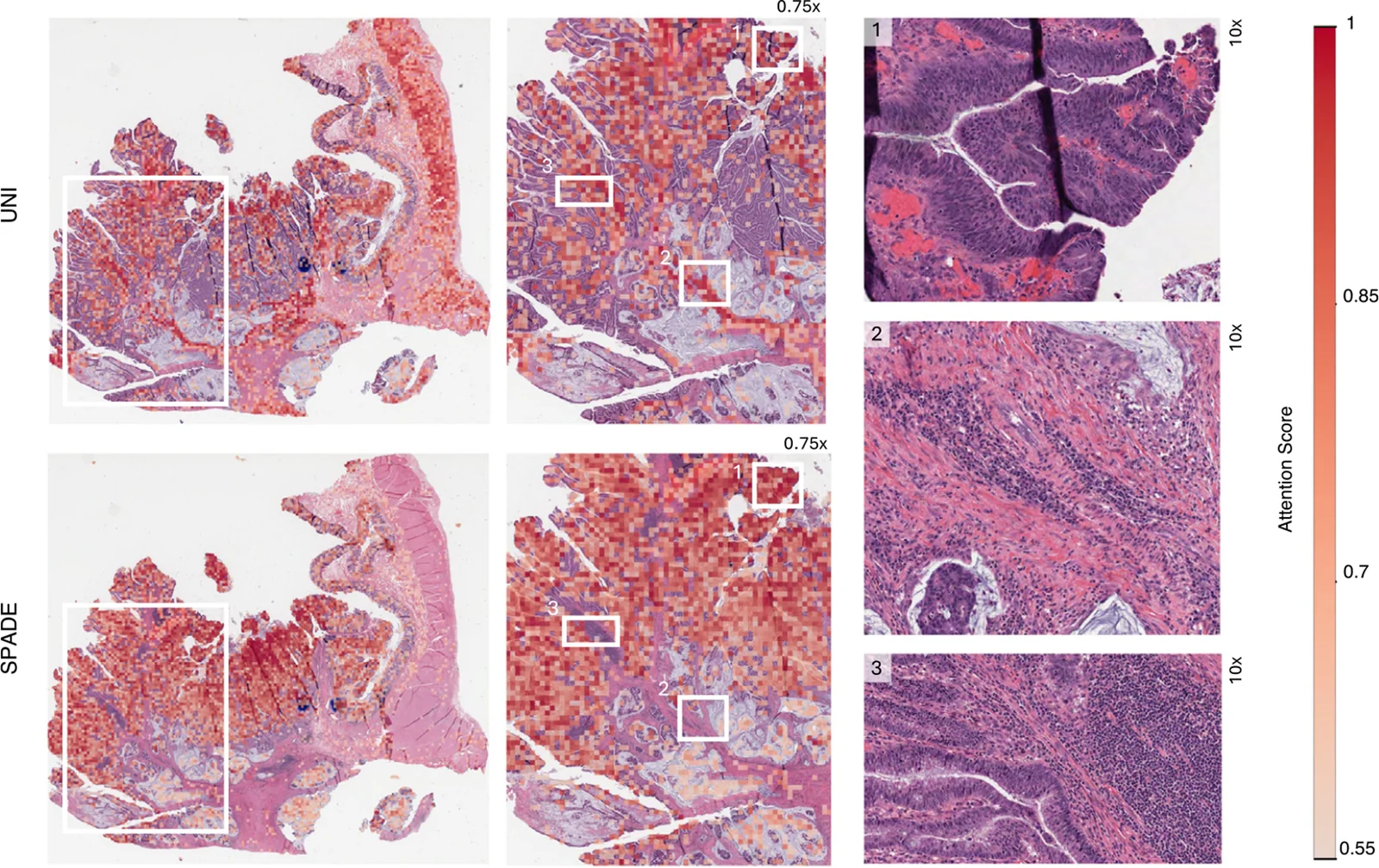
Interpretability of SPADE. Attention scores for a colorectal cancer WSI are visualized as a heatmap for the UNI and SPADE models. The deeper the red color, the higher attention the model puts on that region of the tissue. Sub-crops are selected to show the patterns with high attention at higher magnifications. A pathologist noted differences between UNI and SPADE heatmaps (see Section 4.4.6).

## Data Availability

All data is available publicly with the exception of the biochemical recurrence downstream task, which is private clinical data.

## Appendix A. Datasets

We provide descriptions of the datasets used for the evaluation of SPADE.

### NSCLC

For the non-small cell lung carcinoma (NSCLC) subtyping task, we utilize H&E WSIs from the PLCO and CPTAC datasets to classify cases of lung adenocarcinoma (LUAD) and lung squamous cell carcinoma (LUSC). The PLCO cohort contains 589 slides from 274 patients (LUAD: 171, LUSC: 103). The CPTAC cohort contains 1331 slides from 427 patients (LUAD: 219, LUSC: 208). We perform a patient-level, label-stratified split of both cohorts into training, validation, and test sets with a 70:10:20 ratio. Performance was evaluated using AUCROC and F1-Score.

We also ran a lung cancer survival task using the same PLCO lung cohort. Mortality status from the PLCO study data is used to determine survival, censorship, and duration of survival for this dataset. Out of 274 participants, 213 survived. We performed a patient-level, label-stratified split into training, validation, and test sets with a 70:10:20 ratio. Performance was evaluated using the c-index.

### PANDA

For the prostate cancer ISUP grading task, we utilized 10,616 core needle biopsy cases collected from Karolinska Institute and Rad-boud University Medical Center. All biopsy WSIs were obtained from the Prostate Cancer Grade Assessment (PANDA) challenge. Each biopsy is assigned an ISUP grade on a scale from 1 to 5 if cancerous, while benign biopsies receive a label of 0, resulting in a six-class classification problem. The class distribution is as follows: class 0 (2892), class 1 (2666), class 2 (1343), class 3 (1242), class 4 (1249), and class 5 (1224). Patient identification information was not provided in the dataset, so patient-level splits could not be created. Instead, we perform a label-stratified split into training, validation, and test sets with a 70:10:20 ratio. Performance was evaluated using AUCROC and macro F1-Score.

### Camelyon16

We used the CAMELYON16 dataset, which includes 397 WSIs from sentinel lymph node biopsies, to detect metastases in breast cancer patients. Metastases are categorized into three classes—’negative’ (237), ‘micro’ (80), and ‘macro’ (80)—making this a three-class classification problem. Patient identification information was not provided in the dataset, so patient-level splits could not be created. Instead, we perform a label-stratified split into training, validation, and test sets with a 70:10:20 ratio. Performance was evaluated using AUCROC and macro F1-Score.

### UBC-OCEAN

For ovarian cancer subtype classification, we utilized 513 biopsy samples from the UBC ovarian cancer subtype classification and outlier detection (UBC-OCEAN) competition. Each sample is assigned one out of five histological subtypes of ovarian cancer: class 0 (217), class 1 (42), class 2 (119), class 3 (94), and class 4 (41). Patient identification information was not provided in the dataset, so patient-level splits could not be created. Instead, we perform a label-stratified split into training, validation, and test sets with a 70:10:20 ratio. Performance was evaluated using AUCROC and macro F1-Score.

### Breast

For the breast cancer subtyping task, we used the PLCO Breast dataset. The PLCO cohort consists of 1768 slides from 867 patients. Each sample is categorized into one of three classes - ‘lobular’ (104), ‘ductal’ (657), and ‘other’ (106). We perform patient-level, label-stratified split into training, validation, and test sets with a 70:10:20 ratio. Performance was evaluated using AUCROC and macro F1-Score.

We use the PLCO Breast and TCGA-BRCA datasets for breast cancer survival tasks. Mortality status from the PLCO study data is used to determine survival, censorship, and duration of survival. Out of 867 participants, 552 survived. The TCGA-BRCA dataset consists of 1267 WSIs spanning 1042 patients, with 963 surviving based on disease-specific survival (DSS) status. We perform a patient-level, label-stratified split for both datasets into training, validation, and test sets with a 70:10:20 ratio. Performance was evaluated using the c-index.

We also use the TCGA-BRCA dataset to predict ER, PR, and HER2 receptor status using labels extracted by Kather et al. (2020). Patients were split into 70:10:20 train/validation/test splits at the patient level. Performance was evaluated using AUROC and F1. Cohort sizes and prevalences were: ER: 691 patients (532 positive, 159 negative), PR: 688 (463 positive, 225 negative), and HER2: 680 (105 positive, 576 negative).

### Prostate

For our prostate cancer biochemical recurrence (BCR) task, we use an in-house dataset of radical prostatectomy whole-mount WSIs from UCLA. We also use the TCGA-PRAD dataset, however there are no biochemical recurrence labels provided for this dataset, instead we use progression-free survival parsed by Liu et al. (2018). The in-house dataset spans 161 patients, with one whole-mount WSI selected per patient based on pathology reports. The ratio of patients without BCR versus those with BCR is 135:26. The Gleason grading breakdown of this dataset is: 74 - 3+4 cases, 46 - 4+3 cases, 20 - 4+5 cases, 13 - 3+3 cases, 6 - 4+4 cases, 1 - 3+5 case, and 1 - 5+4 case. The TCGA-PRAD dataset consists of 336 WSIs spanning 322 patients with a progression-free survival rate ratio of 285:37. We perform a patient-level, label-stratified split into training, validation, and test sets with a 70:10:20 ratio for both in-house and TCGA-PRAD datasets. Performance was evaluated using the c-index.

### Uterine

For the uterine cancer survival task, we use the TCGA-UCEC dataset. The dataset consists of 786 images spanning 504 patients, with 445 out of the 504 patients total surviving. We perform a patient-level, label-stratified split into training, validation, and test sets with a 70:10:20 ratio. Performance was evaluated using the c-index.

### Colorectal

For colorectal cancer, we combined the TCGA-READ and TCGA-COAD datasets (TCGA-CRC, N=555) and formed 70:10:20 patient-level splits separately for each target. Labels were extracted from Kather et al. (2020). We evaluated performance using AUROC and F1 score. Class counts and prevalences were: KRAS: 202 positive, 353 negative; BRAF: 51 positive, 504 negative; TP53: 292 positive, 263 negative.

## Appendix B. Model and training

For pretraining training, we employ a weight decay of 1 × 10^−5^ and use the AdamW optimizer with a learning rate of 1 × 10^−4^, along with a cosine decay scheduler. For the slide classification experiments, we utilized a cross-entropy loss. We employed early stopping if the validation loss failed to improve over ten consecutive epochs with a total training epochs of 20. For survival prediction experiments, we used negative log-likelihood loss (NLL).

### ABMIL downstream architecture:

ABMIL architecture used in the downstream experiments consists of three components. First, a 2-layer MLP with 256 or 512 hidden units, layer normalization, ReLu activation, and 0.25 dropout. This is followed by a gated-attention network consisting of 2-layer MLP, with Sigmoid and Tanh activation, respectively, and 0.25 dropout. Finally, a post-attention linear classification layer with 256 or 512 hidden units is applied.

### TANGLE and GigaPath downstream architecture:

For the TANGLE and Gigapath baselines, we employed logistic regression following the open-source instructions provided by the Tangle authors (Jaume et al., 2024b).

### Hyperparameter Tuning and Cross-Validation for Baselines:

To ensure proper comparison between our model SPADE and each baseline model we compare too we performed hyperparameter tuning with Optuna using cross-validation to select the best parameters for each task for each model. For each task, the data were first split into 70% training, 10% validation, and 20% held-out test sets. The 20% test set was kept fixed and was not used during hyperparameter tuning, cross-validation, early stopping, or model selection. The 70% training and 10% validation sets were combined into an 80% development set for five-fold cross-validation, and hyperparameters were selected based on average validation performance across folds. Using the selected hyperparameters, the final model was trained on the original 70% training split, with the original 10% validation split used for epoch selection/early stopping. Final reported performance was computed only on the held-out 20% test-set.

## Appendix C. Clustering

For the proposed 2-step clustering, the size of the uniform sample $D_{i}^{\prime }$ is 1,500,000 patches. The fine-grained K-means is performed over each $D_{i}^{\prime }$ with k=16. A second round of clustering is performed on top of previously found clusters S with k=16.

In the first clustering step, we performed clustering within each tissue type, varying k across a range of values. To determine the optimal number of clusters, we calculated the within-cluster sum of squares (WCSS) and applied the elbow method. The corresponding elbow plot (see Fig. C.6), suggests that the optimal choice is k=16, as improvements in WCSS diminish beyond this point. A secondary drop is observed at k=32, which we included in our ablation studies to assess its impact on downstream task performance.

In the second clustering step, we clustered the centroids obtained prior to form broader, overall clusters. Again, we evaluated values of k using WCSS and generated an elbow plot (see Fig. C.6). In this case, the distinction between k=16 and k=32 is less pronounced. However, given the higher computational cost of running 32 experts, we opted for k=16 to reduce overall model complexity. This approach allowed us to structure the data efficiently while balancing computational cost and cluster granularity.

### C.1. Organ composition of SPADE clusters

To characterize the extent to which the two-step SPADE clustering organizes tissue by organ identity versus by shared morphological phenotype, we quantified the organ composition of each final (second-stage) cluster.

#### Setup.

The first stage produces fine-grained morphological prototypes within each organ. The second stage groups these local prototypes across organs by clustering on the centroids found in the first stage of clustering. For each final cluster j we recorded the number of organs represented, the proportion $p_{j,O}$ of its local prototypes originating from organ O and a normalized organ entropy

(C.1)
$$H_{j}=-\frac{\sum_{O}\;p_{j,O}\;\text{log}\;p_{j,O}}{\text{log}\;O}$$

where O is the total number of organs, $H_{j}$ is bounded between 0 and 1. $H_{j}=0$ indicates a organ-restricted cluster, and $H_{j}$ near 1 indicates a cluster drawn near-uniformly from many organs. Normalization by log O makes the metric comparable across clusters of different sizes

**Table E.8:** One-step clustering for the mixture of experts. We compare the performance of SPADE with 240 data experts using two subtyping tasks. Metrics AUC and F1 are shown as percentages.

	CPTAC Lung		Camelyon	
	AUC	F1	AUC	F1
SPADE	95.9	81.7	97.1	81.9

#### Results.

Final clusters span the full diversity range, from organ-restricted (H=0) to broadly multi-organ (H=0.76). Critically, the partition is not a one-to-one organ-to-cluster mapping, which would means the final 16 clusters follow trivial organ-identify (Fig. C.7).

A single organ can map to multiple final clusters. For example prostate forms two distinct organ-pure clusters, indicating that the clustering separated sub-organ morphological phenotypes rather than collapsing an organ to a single label. Based on our analysis we observe multiple organs frequently share a final cluster. Seven of the sixteen final clusters are multi-organ, drawing on as many as nine organs in each. Additionally, we find that some organs form no dedicated cluster. For example, heart is never organ-pure; it recurs as the dominant organ across several multi-organ clusters, indicating its tissue morphology is repeatedly grouped with that of other organs.

Although 9 of 16 final clusters are organ specific by count, these clusters contain only a minority of the first-stage local clusters (86 of 240, 36%). The majority of local clusters (154 of 240, 64%) are assigned to multi-organ final clusters. In other words, most of the clustering structure is cross-organ, even though slightly more than half of the individual final clusters are organ pure.

The organ-pure clusters are not an artifact of under sampling, they include some of the largest clusters (kidney and breast each comprise 16 local prototypes), indicating that purity reflects genuine morphological distinctiveness of those tissues rather than insufficient data.

**Table F.9:** Comparison of mixture-of-experts (SPADE) to single expert (SPADE-0) for subtyping task. Metrics AUC and F1 are shown as percentages.

	Lung				Prostate				Lymph nodes		Ovarian		Breast			
	PLCO		CPTAC		PANDA		TCGA-PRAD		CAM16		UBC-Ocean		PLCO		TCGA-BRCA	
	AUC	F1	AUC	F1	AUC	F1	AUC	F1	AUC	F1	AUC	F1	AUC	F1	AUC	F1
SPADE-0	**97.3**	92.7	96.1	84.9	**94.5**	**74.9**	74.9	47.9	**96.1**	81	97.3	85.3	77.1	59.5	96.7	93.9
SPADE	96.6	**93.3**	**97.2**	**91.1**	94.2	73.5	**80.5**	**48.8**	95.8	**85.3**	**97.9**	**89.2**	**80.8**	**60.6**	**97.3**	**96.6**

**Table F.10:** Comparison of mixture-of-experts (SPADE) to single expert (SPADE-0) for survival and BCR. c-index values are shown as percentages.

	Prostate		Lung	Uterine	Breast	
	UCLA BCR	TCGA-PRAD	PLCO	TCGA-UCEC	TCGA-BRCA	PLCO
	c-index	c-index	c-index	c-index	c-index	c-index
SPADE-0	**67.7**	**57.4**	49.1	**69.1**	66.8	51
SPADE	65.6	56.6	**52.6**	**69.1**	**71.4**	**54.5**

## Appendix D. Additional interpretability figures

See Figs. D. 8 and D.9.

## Appendix E. Additional experiments, one-step clustering

We implemented a version of SPADE using clusters generated from the first stage of our two-step clustering algorithm, where each organ-specific cohort was clustered with k=16, totaling 240 cluster centroids and, in turn, 240 experts. We report the classification results for two subtyping tasks: Camelyon16 and CPTAC lung.

We observe that data experts trained on cluster centroids obtained with a two-step clustering approach lead to better results compared to a one-step clustering approach (Table E.8). The one-step approach results in a reduction in AUC for the CPTAC dataset, with a decrease of 1.9%, and for the Camelyon dataset, with a decrease of 5.68%. F1 values decreased by 3.03% on the CPTAC dataset. For the Camelyon dataset, F1 values dropped by 12.91%. This can be explained by the fact that one-step clustering leads to cluster centers that are not sufficiently representative of a specific tissue prototype and, therefore, cannot learn a good representation.

## Appendix F. Additional experiments, single expert

To further evaluate the effectiveness of SPADE, we conducted an experiment comparing its performance against a single-expert model (SPADE-0) across subtyping, survival and BCR prediction tasks. As shown in Table F.9, SPADE outperformed SPADE-0 in the vast majority of subtyping datasets. Most notably, SPADE achieved higher F1 scores in seven out of the eight cohorts, often by substantial margins. This demonstrates that incorporating multiple experts tailored to different feature spaces yields more robust and discriminative features for classification.

For continuous risk prediction (survival and BCR, F.10), the results were more nuanced. While SPADE achieved higher c-index scores in the lung and breast cohorts, the single expert SPADE-0 model performed slightly better on both prostate datasets, and the two models tied on TCGA-UCEC. This suggest that while the MoDE routing provides clear advantages for broad classification tasks and several survival endpoints, a single globally aligned expert may sometimes capture sufficient prognostic signal.

**Table G.11:** Cancer subtyping results with Virchow2. Comparison of the mixture-of-experts model (SPADE) to the single-expert variant (SPADE-0), both using Virchow foundation model-derived histology features instead of UNI. The comparison also includes the Virchow2 baseline without any contrastive pretraining. Results are reported in terms of AUC and F1 score (%). The best performance is shown in bold, and the second best is underlined.

	Lung				Prostate				Lymph nodes		Ovarian		Breast			
	PLCO		CPTAC		PANDA		TCGA-PRAD		Camel16		UBC-Ocean		PLCO		TCGA-BRCA	
	AUC	F1	AUC	F1	AUC	F1	AUC	F1	AUC	F1	AUC	F1	AUC	F1	AUC	F1
Virchow + ABMIL	**97.7**	**92.7**	96.3	74.6	**93.9**	**70.9**	79	41.1	**95**	**81.3**	96.5	84.1	77.6	55.8	93.9	96.3
SPADE Virchow2-0 + ABMIL	96.9	84.2	**97.6**	79.4	93.2	67.8	75.3	45.5	93.9	73.3	97	87.8	76.4	53.5	96.7	95.7
SPADE Virchow2 + ABMIL	95.7	85.7	95.9	**86.5**	93.4	69.2	**80.2**	**49.4**	93.1	79.7	**97.6**	**89**	**79.1**	**56.4**	**96.8**	**96.4**

**Table H.12:** Cancer subtyping results comparing MIL aggregation methods. Comparison of SPADE embeddings aggregated using TransMIL versus the baseline ABMIL framework across diverse cancer subtyping tasks. Results are reported in terms of AUC and F1 score (%). The best performance for each dataset is shown in bold.

	Lung				Prostate				Lymph nodes		Ovarian		Breast			
	PLCO		CPTAC		PANDA		TCGA-PRAD		CAM16		UBC-Ocean		PLCO		TCGA-BRCA	
	AUC	F1	AUC	F1	AUC	F1	AUC	F1	AUC	F1	AUC	F1	AUC	F1	AUC	F1
SPADE + Transmil	96.6	82.1	96.4	89.7	89.6	59	72.1	43.9	93.4	82.9	97.3	83.2	72.5	47.9	**97.3**	**97.6**
SPADE + ABMIL	**96.9**	**93.3**	**97.2**	**91.1**	**94.2**	**73.5**	**80.5**	**48.8**	**95.8**	**85.3**	**97.9**	**89.2**	**80.8**	**60.6**	**97.3**	96.6

**Table H.13:** Subset of survival results comparing MIL aggregation methods. Comparison of SPADE embeddings aggregated using TransMIL versus the baseline ABMIL framework across a subset of the 6 survival tasks. Results are reported in terms c-index. The best performance for each dataset is shown in bold.

	Prostate	Lung	Uterine	Breast
	UCLA BCR	PLCO	TCGA-UCEC	TCGA-BRCA
	c-index	c-index	c-index	c-index
SPADE + Transmil	**71.4**	**72.3**	**71**	**71.8**
SPADE + ABMIL	65.6	52.6	69.1	71.4

## Appendix G. Additional experiments, Virchow2 as a backbone

To assess the generality of SPADE across different histology backbones, we repeated the subtyping experiments using Virchow2 features instead of UNI as the image encoder. Table G.11 compares SPADE(Virchow2) to its single-expert variant SPADE(Virchow2)-0 and to the Virchow2 baseline without any contrastive pretraining.

Overall, SPADE demonstrated highly competitive performance, achieving the highest AUC and F1 scores in half of the dataset as well as the top F1 score in the CPTAC cohort. While the robust Virchow2 baseline achieved the highest metrics in three datasets, the improvements SPADE yields in the remaining cohorts highlight that the mixture of experts design and contrastive alignment provide complementary benefits when using alternative foundation models.

Although absolute performance was slightly lower than with the UNI backbone (Table 1), the trends remain consistent, indicating that SPADE’s architecture and training strategy are robust to the choice of foundation model. These findings suggest that SPADE can effectively adapt to diverse histology feature encoders.

## Appendix H. Additional experiments, changing the aggregation head

The tile-level SPADE embeddings are architecture agnostic at the WSI-level for aggregation and can be plugged into alternative MIL frameworks, such as Transmil, CLAM, or DTFD-MIL (Shao et al., 2021; Zhang et al., 2022; Lu et al., 2021). To validate this flexibility, we conducted an experiment replacing our default ABMIL aggregator with TransMIL for downstream WSI-level evaluation across our subtyping tasks.

The Tables H.12, H.13 compare the performance of SPADE when using TransMIL for aggregation versus ABMIL. As demonstrated in these results, SPADE embeddings yield strong, competitive performance regardless of the specific MIL framework. Interestingly, while TransMIL tended to provide greater benefits for survival prediction tasks in our broader testing, ABMIL consistently achieved higher performance across these specific subtyping classification tasks. We initially selected ABMIL as our primary WSI-level representation framework due to its simplicity and wide adoption. The design choice allowed use to better isolate the downstream performance gains specifically attributable to the SPADE embeddings rather than the complexity of the aggregation method itself.

## Appendix I. Additional experiments, UNI+MoDE

To further isolate how much of SPADE’s performance comes specifically from the learned MoDE projections versus the underlying routing and clustering structure itself, we conducted an ablation study. We compared SPADE against a lightweight, model-free baseline termed UNI+MoDE, which replaces SPADE’s learned expert neural networks with a simple centroid-based soft assignment. Both SPADE and this baseline use the exact same frozen UNI image encoder to extract the initial H&E tile-level features.

Unlike SPADE, the UNI+MoDE baseline computes soft-routing weights purely from tile-to-centroid distances and outputs a weighted sum of the original 1024-dimensional UNI features. This produces a weighted mixture of centroid vectors per input patch. As shown in Table I.14, the performance of the pure centroid-routing baselines falls significantly below both the UNI baseline and SPADE. This performance drop suggests that simply clustering and routing the frozen UNI features is insufficient, and that SPADE’s learned MoDE projections are driving the performance.

## Appendix J. Supplementary data

Supplementary material related to this article can be found online at https://doi.org/10.1016/j.media.2026.104197.

**Table I.14:** Cancer subtyping results across UNI, SPADE, and UNI mixture of experts variant. Comparison of subtyping performance across various cancer datasets to isolate the contribution of learned expert projections. The UNI+MoDE baseline uses a simple centroid-based soft routing without learned expert networks. Results are reported in terms of AUC and F1 score (%). The best performance for each dataset is shown in bold, and the second-best is underlined.

	Lung				Prostate				Lymph nodes		Ovarian		Breast			
	PLCO		CPTAC		PANDA		TCGA-PRAD		Camel16		UBC-Ocean		PLCO		TCGA-BRCA	
UNI + ABMIL	**97.1**	**93.3**	**97.3**	90.9	**94.6**	**76.3**	**82.4**	44.8	**96.8**	81.4	**98.1**	87.4	78.1	55.9	96.6	96.2
SPADE + ABMIL	96.9	**93.3**	97.2	**91.1**	94.2	73.5	80.5	**48.8**	95.8	**85.3**	97.9	**89.2**	**80.8**	**60.6**	**97.3**	**96.6**
UNI MoDE + ABMIL	84.4	72.2	91.6	83.7	79.2	43.2	75.2	36.7	66.3	57.2	86.6	57.5	56.3	25.7	87.3	85.6
